# Coordination of Rheb lysosomal membrane interactions with mTORC1 activation

**DOI:** 10.12688/f1000research.22367.1

**Published:** 2020-05-27

**Authors:** Brittany Angarola, Shawn M. Ferguson

**Affiliations:** 1 Department of Cell Biology, Yale University School of Medicine, New Haven, CT, 06510, USA; 2 Department of Neuroscience, Program in Cellular Neuroscience, Neurodegeneration, and Repair, Yale University School of Medicine, New Haven, CT, 06510, USA

**Keywords:** lysosome, signaling, cancer, Rheb, mTOR, membranes

## Abstract

A complex molecular machinery converges on the surface of lysosomes to ensure that the growth-promoting signaling mediated by mechanistic target of rapamycin complex 1 (mTORC1) is tightly controlled by the availability of nutrients and growth factors. The final step in this activation process is dependent on Rheb, a small GTPase that binds to mTOR and allosterically activates its kinase activity. Here we review the mechanisms that determine the subcellular localization of Rheb (and the closely related RhebL1 protein) as well as the significance of these mechanisms for controlling mTORC1 activation. In particular, we explore how the relatively weak membrane interactions conferred by C-terminal farnesylation are critical for the ability of Rheb to activate mTORC1. In addition to supporting transient membrane interactions, Rheb C-terminal farnesylation also supports an interaction between Rheb and the δ subunit of phosphodiesterase 6 (PDEδ). This interaction provides a potential mechanism for targeting Rheb to membranes that contain Arl2, a small GTPase that triggers the release of prenylated proteins from PDEδ. The minimal membrane targeting conferred by C-terminal farnesylation of Rheb and RhebL1 distinguishes them from other members of the Ras superfamily that possess additional membrane interaction motifs that work with farnesylation for enrichment on the specific subcellular membranes where they engage key effectors. Finally, we highlight diversity in Rheb membrane targeting mechanisms as well as the potential for alternative mTORC1 activation mechanisms across species.

## Introduction

The growth of cells is closely matched to changes in the availability of nutrients through a complex set of sensing and signaling mechanisms that regulate the kinase activity of the mechanistic target of rapamycin complex 1 (mTORC1). When nutrients are abundant, activation of mTORC1 results in the phosphorylation of numerous downstream targets to enhance anabolic processes such as nucleotide synthesis and protein translation and suppress catabolic processes such as autophagy and lysosome biogenesis. In many metazoan organisms, growth factor availability is also integrated into the activation of mTORC1 via regulation of the nucleotide-binding state of Ras homolog enriched in brain (Rheb), a small GTPase that binds to and allosterically activates the mTOR kinase when it is part of mTORC1 ^1, 2^.

mTOR is a large (289 kDa) kinase that is a critical component of two complexes: mTORC1 and mTORC2. In this review, we focus on mTORC1, which contains mTOR as well as regulatory subunits including LST8 and Raptor that help with scaffolding, subcellular targeting, and substrate recognition ^3– 7^. Of note, Raptor plays a critical role in targeting mTORC1 to the surface of lysosomes via its interactions with the heterodimeric Rag GTPases ^8, 9^. The functionally distinct mTORC2 is made up of mTOR and LST8, along with a different set of interacting proteins, and does not require Rheb for its activation ^5, 10, 11^.

**Figure 1.**
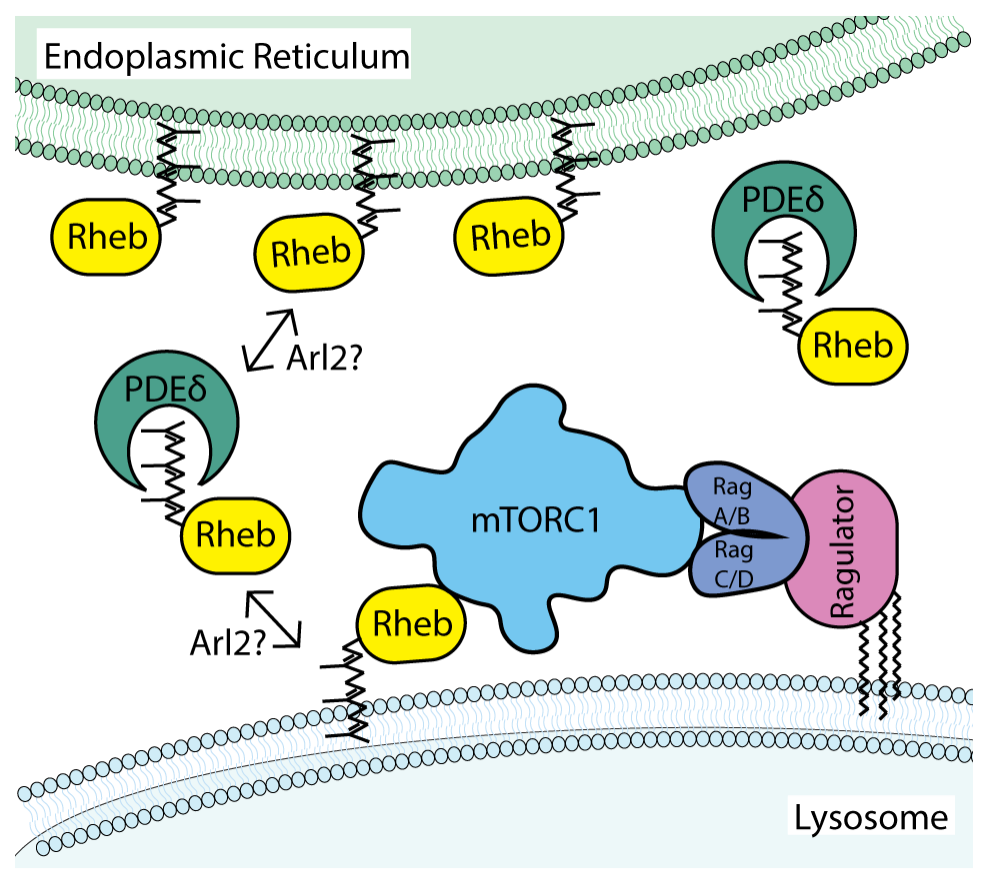
Dynamic interactions of Rheb with lysosomal membranes support the activation of mTORC1 signaling. In this model, farnesylation of Rheb is a major determinant of Rheb subcellular localization. The weak membrane association conferred by farnesylation means that steady-state levels of Rheb on lysosomes are low and influenced by additional factors such as sequestration in the cytoplasm by PDEδ and Arl2-dependent release on target membranes. In contrast to Rheb, the Rag GTPases are enriched on lysosomal membranes through interactions with the pentameric Ragulator complex whose LAMTOR1/p18 subunit is both myristoylated and palmitoylated on an N-terminal glycine with two adjacent cysteines ^21^. Although each of these cysteines represent putative sites of palmitoylation, it remains to be formally established whether they are simultaneously palmitoylated. Furthermore, it was recently reported that LAMTOR1/p18 palmitoylation can be regulated in response to changes in amino acid availability ^22^. Although simplified in this schematic diagram, mTORC1 is a dimer and can thus potentially engage a total of six GTPases on the surface of lysosomes (two Rag heterodimers that can be made up of different pairwise combinations of RagA or RagB with RagC or RagD plus two Rheb/RhebL1) ^1, 23^. mTORC1, mammalian target of rapamycin complex 1; PDEδ, δ subunit of phosphodiesterase 6; Rheb, Ras homolog enriched in brain.

The cytoplasmic surface of lysosomes is the major site within cells where mTORC1 activation occurs ^8, 9^. mTORC1 activation is highly regulated and dependent on the convergence and integration of multiple signals relaying growth factor and nutrient availability to ensure that the cell growth-promoting activity of mTORC1 is matched to ongoing changes in environmental status ^8, 12– 18^. GTP-bound Rheb subsequently binds and activates mTORC1. This model requires mechanisms to regulate the abundance and nucleotide state of both Rag and Rheb GTPases on the surface of lysosomes. However, in contrast to the Rags, which are enriched on lysosomes via their interactions with the lysosome-localized Ragulator complex ^8, 9, 18, 19^, there has long been a mismatch between the widespread belief that Rheb abundantly resides on the surface of lysosomes and minimal direct evidence to support such a localization. This review highlights evidence that reconciles differences between Rheb steady-state localization and function via a model wherein transient interactions of Rheb with lysosomal membranes support mTORC1 activation ( Figure 1).

## Rheb is a critical link between growth factor receptor signaling and mTORC1 activation

Rheb was originally identified as a gene whose expression was upregulated by multiple stimuli that increased neuronal activity in the rat brain ^20^. Although it was discovered in this neuronal context, the broad expression pattern of Rheb suggested functions with relevance beyond the adaptive response of neurons to changes in activity ^20^. Indeed, a major role for Rheb in the regulation of cell growth was subsequently revealed by genetic studies in *Drosophila*, which identified a critical role for Rheb in promoting protein translation by activating mTORC1 signaling ^24– 27^. It was demonstrated that this function of Rheb is inhibited by the tuberous sclerosis complex (TSC), which acts as a GTPase-activating protein (GAP) for Rheb and thereby suppresses the ability of Rheb to activate mTORC1 by converting Rheb from the GTP- to GDP-bound state ^25, 28– 32^. Growth factor signaling promotes Rheb-dependent activation of mTORC1 via AKT-dependent phosphorylation of the TSC2 subunit of TSC, which creates a 14–3–3 binding site on TSC2 and results in the sequestration of TSC2 away from lysosomes ^33– 40^. Loss-of-function mutations in the human TSC1 and TSC2 genes cause tuberous sclerosis, a disease characterized by mTORC1 hyperactivation and a variable constellation of distinct tumors as well as seizures and psychiatric disabilities ^41^. Rheb has also been broadly implicated in a wide range of diseases including cancer, diabetes, aging, neurodegeneration, epilepsy, and autism, where excessive mTORC1 signaling plays a role ^18, 42^. The importance of mTORC1 over-activation in multiple disease states has motivated the development of pharmacological strategies for Rheb inhibition ^43^.

Interestingly, given the abundant evidence demonstrating that regulating Rheb activity is critical in both health and disease, no guanine nucleotide exchange factor (GEF) has been identified for Rheb. Although translationally controlled tumor protein (TCTP) was proposed to act as a Rheb GEF, this conclusion was subsequently challenged ^44– 46^. The ubiquitin E3 ligase known as PAM was also proposed to be a Rheb GEF, but there have not been follow-up studies to substantiate that this is physiologically relevant ^47^. In the absence of a GEF, it is thought that a relatively low nucleotide affinity allows Rheb to spontaneously exchange GDP for GTP ^45, 46^. The nucleotide state of Rheb is thus predicted to reflect the cellular GTP/GDP ratio when growth factor-dependent signaling has suppressed the GAP activity of TSC. As GTP generally predominates over GDP in cells, this results in a predominantly active state for Rheb ^29, 30^. In this active, GTP-bound state, Rheb can bind to mTORC1 and trigger long-range conformational changes that result in a reorganization and activation of the mTOR kinase domain ^1^. Although this allosteric regulation of mTORC1 by Rheb is mediated by the GTP-bound state of Rheb, it appears that the affinity of the interaction between Rheb and mTORC1 is weak and/or transient such that the endogenous complex between Rheb and mTORC1 cannot readily be isolated from cells by conventional affinity purification methods ^30, 48^. Rheb thus differs from closely related small GTPases such as Ras proteins that bind to effectors such as Raf with high affinity ^49, 50^. Looking to the future, there is still a need to elucidate how mTOR conformational changes that are induced by Rheb are either maintained or terminated and how such steps contribute to the regulation of mTORC1 signaling.

## Rheb and RhebL1 function redundantly in the activation of mTORC1

In addition to Rheb, whose role in mTORC1 activation is well-characterized, mammalian genomes also contain a gene for a related GTPase called *Rheb-like 1* ( *RhebL1*, also known as *Rheb2*) that shares only ~52% amino acid identity with Rheb but is also able to activate mTORC1 when over-expressed ^51, 52^. Although their redundant ability to activate mTORC1 signaling has long been established by over-expression approaches, considerably less attention has been paid to physiological roles of RhebL1 and most studies have simply referred to Rheb as the activator of mTORC1. However, even though Rheb knockout (KO) mice have an embryonic lethal phenotype and exhibit defects in the activation of mTORC1, embryonic fibroblasts from these mice still grew in culture ^53– 55^. These cells maintained a basal level of mTORC1 activation but were markedly deficient in acutely re-activating mTORC1 signaling when starved and then stimulated with growth factors ^53, 54^. Likewise, the conditional KO of Rheb reduced but did not eliminate mTORC1 signaling in the mouse brain ^56^. The presence of even some limited mTORC1 signaling following Rheb depletion suggested either that there might be a novel way to activate mTORC1 that was distinct from the Rheb-dependent mechanism or that RhebL1 was able to fulfill this function. Unambiguously establishing a requirement for Rheb in activating mTORC1 was complicated by the essential role for mTORC1 signaling in promoting cell growth, which precluded the possibility of generating and maintaining animals or cells that completely lack such activity. However, the combined use of CRISPR-based mutagenesis and siRNA-mediated depletion was recently used to achieve highly efficient but transient depletion of both Rheb and RhebL1 in human cells ^57^. This dual strategy to acutely deplete both Rheb and RhebL1 was required to fully inhibit mTORC1 signaling, and re-expression of either Rheb or RhebL1 restored mTORC1 signaling in the double depleted cells. These observations established that Rheb and RhebL1 can independently activate mTORC1 in human cells. This conclusion is further supported by *in vitro* assays where Rheb and RhebL1 were each able to activate mTORC1 ^58^.

## Rheb farnesylation is essential for mTORC1 activation

**Figure 2.**
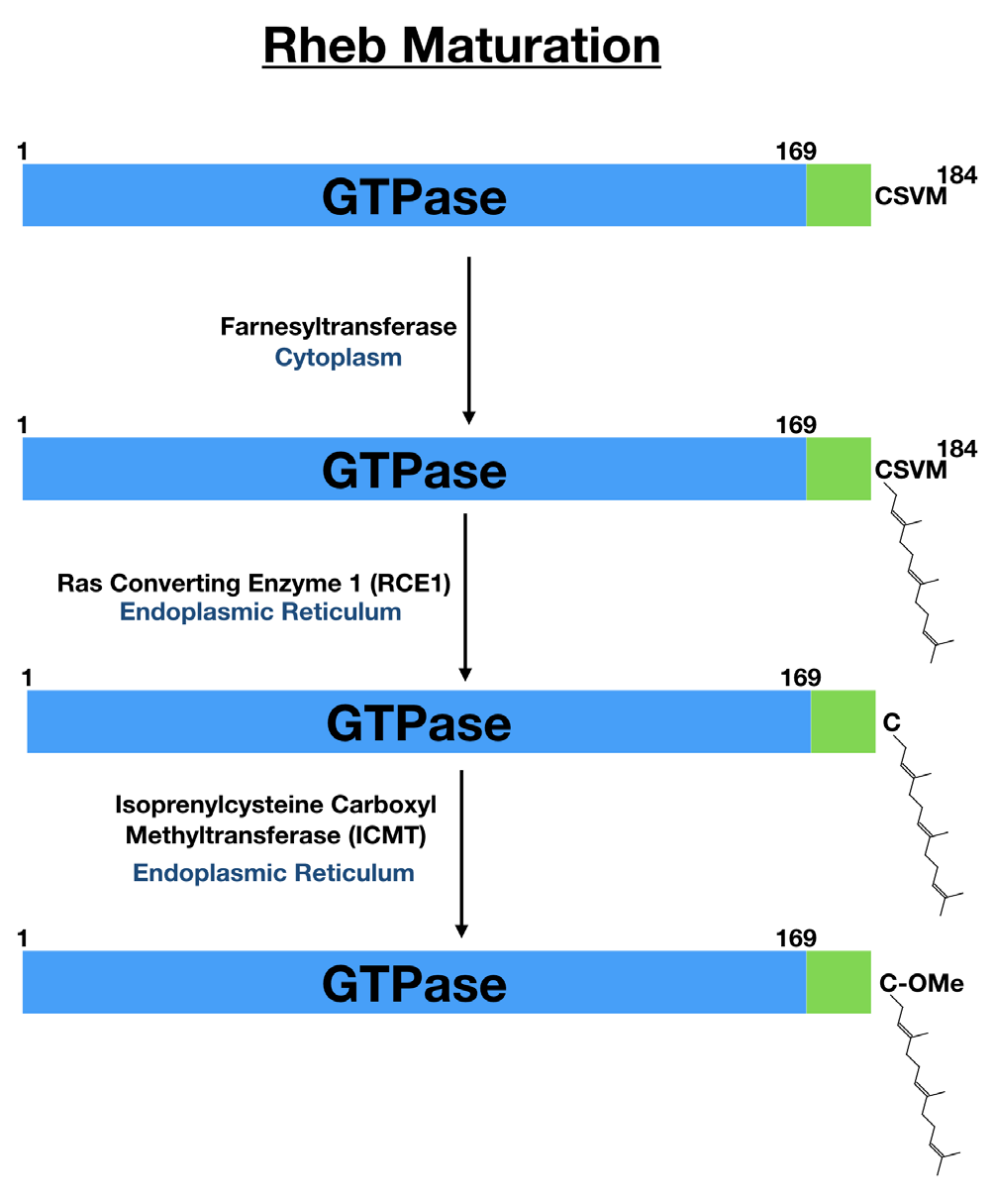
Rheb maturation takes place through a series of modifications to the C-terminal CaaX motif. Human Rheb is translated as a 184-amino-acid protein. The first 169 amino acids contain the GTPase domain (blue). This is followed by a short linker (green) that is sometimes referred to as the hypervariable region. The last four amino acids fit the consensus for a CaaX motif (cysteine followed by two aliphatic amino acids and with flexibility in the final position). This CaaX motif is subsequently farnesylated on the cysteine followed by trimming of the final three amino acids by the ER-localized Ras converting enzyme 1 (RCE1) and methylation of the newly exposed carboxyl group by isoprenylcysteine carboxyl methyltransferase (ICMT, also at the endoplasmic reticulum). Rheb, Ras homolog enriched in brain.

The coincidence detection model whereby nutrient and growth factor-dependent signaling are integrated on the surface of lysosomes by Rags and Rheb to support mTORC1 activation requires a mechanism for targeting Rheb to lysosomes. Human Rheb is a relatively simple protein that consists of just 184 amino acids that encode a GTPase domain within the first 169 amino acids followed by a short alpha helical linker known as the hypervariable region and finishing with the sequence CSVM, which conforms to the CaaX motif that is a signal for farnesylation ( Figure 2) ^20, 50, 59^. Farnesylation of the Rheb C-terminal CaaX motif is part of a multi-step process of post-translational modification ( Figure 2). Following addition of the 15-carbon isoprenyl-based farnesyl group by the cytoplasmic farnesyl transferase on the cysteine within the CaaX motif of Rheb, the Rheb C-terminus is further processed at the endoplasmic reticulum by Ras-converting enzyme 1 (RCE1), which removes the final three C-terminal amino acids, and isoprenylcysteine carboxyl methyltransferase (ICMT), which methylates the C-terminus ^60– 62^. These modifications, which are shared by other proteins that contain a CaaX motif, are predicted to enhance the hydrophobicity and thus membrane binding of the Rheb C-terminus ^63, 64^.

Even though farnesylation is not required for the ability of Rheb to activate mTORC1 signaling *in vitro*, Rheb mutants lacking the CaaX motif cannot activate mTORC1 signaling in cells ^31, 57, 65^. This is thought to reflect a critical role for farnesylation in controlling the subcellular localization of Rheb. However, farnesylation and associated C-terminal modifications are expected to support only weak and therefore transient membrane binding of Rheb ^63, 64, 66^. This weak membrane interaction that lacks specificity for a particular organelle is seemingly at odds with models that imply an enrichment of Rheb at the surface of lysosomes for the purpose of mTORC1 activation.

## Rheb is most prominently enriched on the endoplasmic reticulum

Although a broadly accepted model for mTORC1 activation is centered on lysosomes ^14, 15, 18, 67^, early localization studies based on Rheb over-expression yielded evidence of farnesylation-dependent localization of Rheb to poorly defined “endomembranes” and/or to the endoplasmic reticulum and Golgi rather than a distinct enrichment on lysosomes ^60, 61, 65^. It was possible that the lack of distinct lysosome localization for Rheb in these studies reflected an artifact of the over-expression of tagged Rheb proteins or a limitation of the imaging resolution. Indeed, another study which used immunofluorescence confocal microscopy with a Rheb antibody to localize the endogenous Rheb protein reported Rheb enrichment on lysosomes but still lacked the resolution to optimally resolve individual lysosomes ^37^. However, a more recent study which used the same anti-Rheb antibody and which had improved resolution of lysosome morphology instead found that endogenously expressed Rheb predominantly localized to the endoplasmic reticulum rather than to lysosomes ^57^. As both studies employed robust controls for establishing antibody specificity, resolution differences in these microscopy studies could potentially explain the differing conclusions regarding the presence or absence of lysosome enrichment for Rheb, as the extensive contacts between endoplasmic reticulum and lysosomes raise challenges for distinguishing between these organelles in the crowded perinuclear region of cells. As an alternative approach to defining the key determinants of Rheb localization and function, mutagenesis of putative targeting motifs within the Rheb C-terminus revealed that although farnesylation is essential for Rheb-dependent mTORC1 activation, sequences within the immediately upstream hypervariable region were not conserved between Rheb and RhebL1 and could even be replaced by an unrelated sequence derived from H-Ras ^57^. In contrast, the last 15 amino acids of Rheb were previously reported to act as a lysosome-targeting signal that could be transplanted to other proteins in the mTORC1 pathway ^8^. However, these seemingly disparate interpretations of the role played by the Rheb C-terminus in controlling Rheb localization and function can be resolved by a model wherein weak membrane interactions mediated by the farnesylated C-terminus allow Rheb to transiently visit lysosomes and thus increase opportunities for encountering mTORC1 that has been recruited there by interactions with the Rags. Such encounters could be achieved without requiring significant enrichment of Rheb at lysosomes. One study proposed a role for microspherule protein 1 (MCRS1) in recruiting Rheb to lysosomes ^68^. However, this conclusion has yet to be independently corroborated. In addition, mechanisms have not been defined for the proposed regulated interactions between MCRS1 and Rheb or for MCRS1 interactions with lysosomes. Although it has been challenging to visualize Rheb enrichment on lysosomes in human cell lines, it was very recently reported that the *Caenorhabditis elegans* RHEB-1 protein exhibits such localization ^69^. This new observation raises questions about the mechanisms that target Rheb to lysosomes in this organism and how they might be regulated for the control of mTORC1 signaling in this organism, which lacks TSC.

The functional significance, if any, of the localization of Rheb to the endoplasmic reticulum remains unknown. It has been proposed that endoplasmic reticulum-localized Rheb activates mTORC1 on lysosomes via contact sites between these organelles ^70^. However, constitutive anchoring of Rheb on the endoplasmic reticulum via a transmembrane domain did not restore mTORC1 signaling when introduced into Rheb+RhebL1-depleted cells ^57^. Endoplasmic reticulum localization of Rheb might not be of fundamental functional importance but may instead simply match expectations for a farnesylated protein that lacks other major determinants of subcellular targeting. This is supported by observations that farnesylation of green fluorescent protein (GFP) via the addition of a CaaX motif to its C-terminus also results in endoplasmic reticulum localization ^71^. Even though evidence is lacking for a model wherein endoplasmic reticulum-localized Rheb reaches across contact sites to activate mTORC1 on lysosomes, such contact sites were recently shown to influence mTORC1 signaling via oxysterol-binding protein (OSBP)-mediated cholesterol transport from the endoplasmic reticulum to lysosomes and are thus of relevance for mTORC1 signaling ^72^. It was also proposed that Golgi-localized Rheb activates mTORC1 on lysosomes via interactions that occur across Golgi–lysosome contact sites ^73^. However, these conclusions were based on the analysis of an over-expressed chimeric protein that artificially targeted Rheb to the Golgi, and it remains to be established that mTORC1 activation by the endogenous Rheb or RhebL1 proteins is dependent on sites of Golgi–lysosome contact.

The importance of fine-tuned Rheb subcellular targeting mechanisms for mTORC1 activation is highlighted by observations that mTORC1 signaling becomes insensitive to nutrient inputs when cells over-express Rheb ^51, 65^. This presumably reflects the fact that when Rheb levels are very high, mTORC1 activation no longer requires the highly regulated mechanisms that are normally required to facilitate encounters between Rheb and mTORC1 on the surface of lysosomes.

## Comparison of membrane targeting mechanisms between Rheb and other Ras family members

Comparison of Rheb with other members of the Ras family reveals important differences in their membrane interaction and subcellular localization mechanisms. Like Rheb, H-Ras and K-Ras-4B are small GTPases whose protein sequences comprise the GTPase domain, a short linker, and the C-terminal CaaX motif. Nonetheless, both of these proteins are highly enriched at the plasma membrane ^74, 75^. This is because of conserved sequences in the hypervariable domains of H-Ras (palmitoylated cysteines) and K-Ras-4B (polybasic region) which cooperate with farnesylation to target these proteins to the plasma membrane. Meanwhile, the hypervariable region of human Rheb does not contain any known motifs to confer further specificity or affinity for interactions with specific intracellular membranes. Thus, even though evolution has selected for simple additional mechanisms for targeting other members of the human Ras superfamily to specific subcellular membranes, Rheb does not harbor additional targeting signals that lead it to be enriched on lysosomes. The fact that evolution did not select for additional membrane-targeting signals in Rheb suggests that weak farnesylation-dependent membrane interactions represent a sufficient strategy for Rheb-dependent mTORC1 activation.

## Support for a lysosome-localized function for Rheb

Even though high-resolution visualization of Rheb enrichment on the surface of lysosomes in mammalian cells has been challenging to achieve and there is no defined lysosome-targeting motif within Rheb, there are still several strong arguments that support a lysosome-localized function for Rheb in the activation of mTORC1 signaling.

First, it is well established that mTORC1 recruitment to lysosomes via highly regulated interactions between Raptor and the Rag GTPases is a critical determinant of mTORC1 activation ^9, 18, 76, 77^. It is also clear that Rheb and RhebL1 function redundantly and are essential for mTORC1 activation in human cells ^57^. If mTORC1 is activated at lysosomes and requires Rheb/RhebL1 for this activation, then it follows that Rheb/RhebL1 should at least visit lysosomes transiently to fulfil such a function.

Second, the TSC accumulates on the cytoplasmic surface of lysosomes in response to cell stress and nutrient deprivation ^37, 78– 80^. The presence of this GAP for Rheb on lysosomes argues that lysosomes are central to Rheb regulation. This conclusion is further supported by the observation that constitutive targeting of TSC to lysosomes suppressed mTORC1 signaling ^37^.

Third, the *Rheb* gene is broadly conserved across diverse branches of the eukaryotic family tree. However, the CaaX motif that supports Rheb C-terminal farnesylation is not uniformly present ^81^. Instead, Euglenozoa have a Rheb that lacks C-terminal farnesylation and is instead predicted to interact with membranes via an N-terminal FYVE domain. Although this FYVE domain has not yet been well-characterized, FYVE domains are best known for their ability to bind to phosphatidyl-3-phosphate (PI3P) and thus recruit proteins that harbor them to endolysosomal membranes that are enriched in this lipid ^82, 83^. The presence of a FYVE domain in the Euglenozoa Rheb is thus suggestive of a function in the endolysosomal pathway. Likewise, the Rheb C-terminal CaaX motif in Cryptista is accompanied by an N-terminal PX domain, another protein module that confers weak PI3P interactions ^81^. These putative PI3P interaction mechanisms for Rheb proteins point to the importance of weak membrane interactions and suggest a preference for endolysosomal membranes. Even for human Rheb, the requirement for C-terminal farnesylation can be overcome by providing another weak mechanism for membrane association such as that conferred by N-terminal myristoylation ^57^. These studies also indicate that although farnesylation of Rheb is essential for its ability to activate mTORC1, the farnesylated C-terminus is not directly part of the activation mechanism. The lack of a direct requirement for Rheb farnesylation in mTORC1 activation was also supported by *in vitro* assays where recombinant Rheb which lacks farnesylation still activated mTORC1 ^58^.

## Rheb-independent mechanisms for mTORC1 activation in budding yeast

Analysis of Rheb protein sequences across diverse organisms yields a diversity of putative endo-lysosomal targeting mechanisms. Although it is interesting to interpret this information in the context of what is known about human mTORC1 activation, additional functions for Rheb and alternative mechanisms for mTORC1 activation should also be considered. Phylogenetic analyses have predicted that some organisms contain genes for mTORC1 subunits (mTOR, Raptor, LST8) and yet lack TSC1, TSC2, and/or Rheb ^84– 86^. It remains to be determined to what extent this reflects limitations in gene annotation versus the genuine existence of alternative mechanisms for mTORC1 activation, as this pathway has not been extensively studied in many of the organisms whose sequenced genomes provided information for phylogenetic analyses. However, *Saccharomyces cerevisiae*, an exceedingly well-studied model organism, lacks both *TSC1* and *TSC2* genes ^85– 87^. Furthermore, in spite of considerable literature focused on TORC1 signaling in budding yeast, there is currently no published demonstration that the Rheb homolog (Rhb1) is required for TORC1 signaling, although it has been implicated in the regulation of amino acid transporters ^87^. The lack of evidence for Rheb-dependent TORC1 activation in *S. cerevisiae* raises questions about how Rheb-independent TORC1 activation is achieved in this organism. Such activation still takes place on the surface of the vacuole (yeast lysosome), is regulated by nutrient availability, and is dependent on interactions with the Gtr1 and Gtr2 GTPases which are homologous to the Rags ^88^. Interestingly, it was recently revealed that TORC1 oligomerizes on the surface of the vacuole in response to glucose deprivation and that this is required for TORC1 inactivation ^89^. It remains to be determined whether similar oligomerization-based regulatory mechanisms for mTORC1 take place on the surface of mammalian lysosomes.

## PDEδ binds Rheb in the cytoplasm

**Figure 3.**
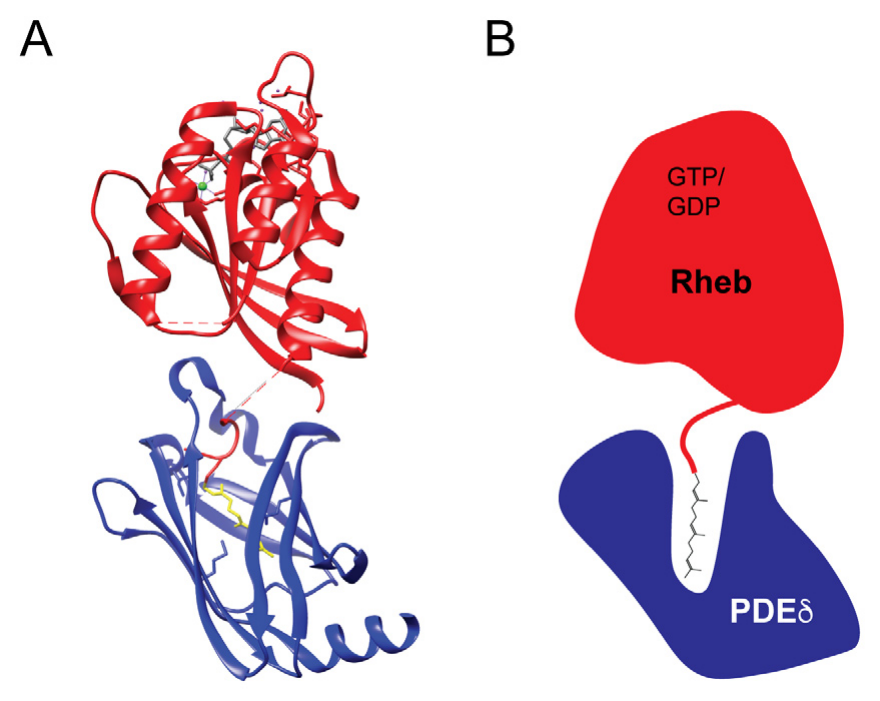
Rheb C-terminal farnesylation allows for interactions with PDEδ. ( **A**) A structure (3T5G) of the complex formed between Rheb (red) and PDEδ (blue) reveals that the interaction is largely independent of contacts between the Rheb and PDEδ proteins and is instead based on the insertion of the Rheb farnesyl group (yellow) deep into a hydrophobic cavity within PDEδ ^91^. Additional features include GDP bound to Rheb in gray, Mg2 ^+^ in green, and water molecules in purple. ( **B**) Schematic diagram of the Rheb–PDEδ heterodimer which highlights the insertion of the farnesyl group from Rheb deep into the hydrophobic cavity at the core of PDEδ. PDEδ, δ subunit of phosphodiesterase 6; Rheb, Ras homolog enriched in brain.

Dynamic interactions between Rheb and lysosomal membranes raise questions about factors that regulate its interactions with membranes. In contrast to the Rab subfamily of GTPases that preferentially bind to GDP dissociation inhibitor (GDI) and are thus stabilized in the cytoplasm when in the GDP-bound state, Rheb and RhebL1 do not interact with a solubilizing factor in a manner that depends on their nucleotide state ^90^. Instead, Rheb and RhebL1 (as well as other Ras family members and many other prenylated proteins) interact in the cytoplasm with phosphodiesterase δ (PDEδ) ^91– 93^. PDEδ was named based on its discovery as the δ subunit of phosphodiesterase 6, which degrades cGMP in rod and cone cells of the retina ^94^. This interaction of PDEδ with the farnesylated PDE6 protein was later found to be generalizable to other farnesylated proteins such as Rheb, other Ras family members, and many other proteins that undergo C-terminal prenylation (farnesylation or geranylgeranylation) ^95– 97^. An explanation for the ability of PDEδ to bind to so many structurally distinct proteins whose only common feature is C-terminal prenylation came from a crystal structure of Rheb in complex with PDEδ, which revealed that the interaction is mediated by the insertion of the farnesyl group deep within a hydrophobic cavity on PDEδ without the need for any major contacts between the rest of the Rheb protein and PDEδ ( Figure 3). Owing to the minimal contact between the protein part of Rheb and PDEδ, this mode of interaction is insensitive to the Rheb nucleotide state ^91^. A role for PDEδ in the regulation of Rheb function is consistent with the inhibition of mTORC1 signaling following treatment of cells with deltarasin, a drug that competes with Rheb for binding to the hydrophobic pocket of PDEδ ^57, 98^. However, because of the many prenylated protein partners of PDEδ whose function might also be perturbed by deltarasin, multiple factors beyond just the disruption of Rheb–PDEδ interactions could contribute to the effect of deltarasin on mTORC1 signaling.

The displacement of prenylated proteins such as Rheb from PDEδ is stimulated by Arl2, a small GTPase ^91, 99– 101^. Although a comparable ability of Arl3 to promote dissociation of Rheb from PDEδ was previously reported ^91^, it now appears that Arl3 function is most physiologically important at cilia, where it promotes the release of myristoylated cargos from another set of solubilizing factors known as UNC119a and UNC119b ^102^. Meanwhile, the relevance of Arl2 for prenylated protein displacement from PDEδ is supported by the observation that Arl2 depletion from cells is sufficient to suppress the release of K-Ras from PDEδ ^92^. This Arl2-dependent re-targeting mechanism is thought to prevent entropic randomization of the distribution of prenylated client proteins and was recently suggested to play a role in the regulation of Rheb-dependent mTORC1 activation ^103^. At the moment, relatively few studies have investigated the interaction between PDEδ and Rheb, the impact of this interaction on Rheb subcellular localization, and the overall consequences of PDEδ-dependent mechanisms for mTORC1 signaling. Further research is required to define how regulation of the interactions between Rheb and PDEδ is integrated with the overall control of mTORC1 signaling by the rest of the cellular machinery dedicated to sensing and responding to changes in nutrient and growth factor availability. If the importance of PDEδ for mTORC1 signaling is solidified by additional studies, then it will become critical to answer questions about the identity, regulation, and subcellular localization of the Arl2 GEF(s).

## Summary

Although Rheb is not highly enriched at lysosomes, multiple lines of evidence coming from studies of the subcellular localization of Rheb targets and regulatory proteins, rescue assays in Rheb/RhebL1-depleted cells, and phylogenetic analyses support a model wherein Rheb transiently visits the surface of lysosomes in order to activate mTORC1. In human Rheb, the weak membrane interactions that are conferred by farnesylation fulfil this role. In contrast to expectations for other small GTPases that exhibit more robust enrichment on target membranes and well-defined mechanisms for supporting such localization, the dynamic membrane-targeting ability conferred by Rheb C-terminal farnesylation coupled with weak interactions between mTORC1 and Rheb may be sufficient to facilitate the initial encounter with lysosome-localized mTORC1 via a reduction of dimensionality but still allow activated mTORC1 to leave lysosomes in order to phosphorylate downstream targets elsewhere in the cell. Although much has been discovered with respect to mechanisms of Rheb-dependent mTORC1 activation, it remains unclear whether Rheb must remain bound to mTORC1 in order to maintain the kinase activity of TOR and whether additional regulatory mechanisms act through Rheb to terminate this signaling.
